# The Treatment of Acne Vulgaris

**Published:** 1911-09

**Authors:** 


### The Treatment of Acne Vulgaris

Burke (*Pennsylvania Medical Journal,* March, 1911) states that with stock vaccines he has not noticed any benefit in the treatment of acne vulgaris. After briefly alluding to the favoring conditions produced by constipation, anemia, and sluggish circulation, he advises puncturing and evacuation of all pustules by means of a comedone extractor, the latter instrument being employed for all comedones large enough to be seen. The face is scrubbed vigorously every night before retiring with green soap and hot water; thereafter rinsed with cold water and greased with an ointment made up of:

℞ Beta-naphthol, 5 per cent;
Sulphur prec., 25 per cent;
Sapo viridis, 35 per cent;
Adeps lanæ, 35 per cent.

This ointment is allowed to remain on for from fifteen minutes to an hour and then wiped off. In very obstinate cases, he advises *x*-ray under careful supervision.—*Therapeutic Gazette.*

